# Heat stress induces spikelet sterility in rice at anthesis through inhibition of pollen tube elongation interfering with auxin homeostasis in pollinated pistils

**DOI:** 10.1186/s12284-018-0206-5

**Published:** 2018-03-12

**Authors:** Caixia Zhang, Guangyan Li, Tingting Chen, Baohua Feng, Weimeng Fu, Jinxiang Yan, Mohammad Rezaul Islam, Qianyu Jin, Longxing Tao, Guanfu Fu

**Affiliations:** National key Laboratory of Rice Biology, China National Rice Research Institute, Hangzhou, 31000 China

**Keywords:** Auxins, Heat stress, *Oryza sativa*, Pollen tube elongation, Reactive oxygen species, Peroxidase

## Abstract

**Background:**

Pollen tube elongation in the pistil is a key step for pollination success in plants, and auxins play an important role in this process. However, the function of auxins in pollen tube elongation in the pistil of rice under heat stress has seldom been previously reported.

**Results:**

Two rice genotypes differing in heat tolerance were subjected to heat stress of 40 °C for 2 h after flowering. A sharp decrease in spikelet fertility was found in the Nipponbare (NPB) and its mutant High temperature susceptible (HTS) under heat stress, but the stress-induced spikelet sterility was reversed by 1-naphthaleneacetic acid (NAA), especially the HTS. Under heat stress, the pollen tubes of NPB were visible in ovule, while those of HTS were invisible. However, we found the pollen tubes in ovule when sprayed with NAA. During this process, a significant increase in indole-3-acetic acid (IAA) and reactive oxygen species (ROS) levels was found in the pistil of heat-stressed NPB, while in heat-stressed HTS they were obviously decreased. Additionally, the peroxidase (POD) activity in pistil of NPB was significantly decreased by heat stress, whereas there was no difference between the heat-stressed and non-heat-stressed pistils of HTS.

**Conclusion:**

It was concluded that the enhancement of heat tolerance in plants by NAA was achieved through the increase of the levels of auxins, which prevented the inhibition of pollen tube elongation in pistil, and the crosstalk between auxins and ROS, which might be involved in this process. In addition, POD might be a negative mediator in pollen tube elongation under heat stress due to its ability to scavenge ROS and degrade auxin.

**Electronic supplementary material:**

The online version of this article (10.1186/s12284-018-0206-5) contains supplementary material, which is available to authorized users.

## Background

High temperature stress is one of the most limiting factors in crop production worldwide as a result of global warming (Fu et al. 2015a; Zhang et al. 2017). It has been suggested that, at least in the cases of wheat, maize, rice and barley, a negative correlation exists between worldwide crop yields and increased temperatures (Reilly and Fuglie 1998; Yu et al. 2012; Karunaratne and Wheeler 2015; Li et al. 2016). For these crops, recent climate warming has resulted in annual combined loss of about megatons (Mt) of products (Peng et al. 2004; Challinor et al. 2014). Rice plants are most sensitive to high temperature during the flowering stage (Satake and Yoshida 1978). High temperature at this stage significantly decreases spikelet sterility in rice, or can even lead to no harvest (Satake and Yoshida 1978; Fu et al. 2012), which can be mainly ascribed to inhibitions of anther dehiscence, pollen sterility and failed germination on the stigma (Jagadish et al. 2010, 2014; Sebastian et al. 2017). In addition, cessation of pollen tube elongation in the pistil is another important factor causing pollination failure under heat stress (Karapanos et al. 2010; Snider et al. 2011), which has seldom been previously documented.

Pollen tube elongation is a key step for successful pollination in plants, including rice, during which the respiration rate, carbohydrate and protein metabolism and phytohormone production are clearly increased to provide the energy (Taylor and Hepler 1997; Kovaleva et al., 2004; Wang et al., 2010; Selinski et al. 2014). These dramatic metabolism activities in pollinated pistil have been reported in relation to auxin, which are beneficial for competing for nutrients, sugars and water (Pan et al. 2004; Lu et al. 1988). Also, as one of the most important hormones, auxins play critical roles in promoting cell elongation and division and pollen development (Feng et al. 2006; Cecchetti et al. 2008; Sakata et al. 2010), as well as the reproductive tissues, including pollen, which contain high concentrations of auxins (Feng et al. 2006; Cecchetti et al. 2008; Chen and Zhao 2008). Moreover, since exogenous auxins can stimulate in vitro pollen tube growth (Chen and Zhao 2008; Wu et al. 2008), it can be assumed that auxin signaling may play a potential role in regulating the elongation of pollen tubes. This finding was supported by studies on the pollen-specific auxin efflux carrier *PIN8* indicating that its mutant pollen had a reduced germination ratio compared to wild-type, whereas *PIN8* overexpression in pollen increased the resistance of pollen tube elongation to 1-N-naphthylphthalamic acid (NPA) (Ding et al. 2012). The results of Wu et al. (2008) indicated that auxin regulated the pollen tube elongation and its direction by secreting vesicles in pollen tube, thus enhancing PM H^+^-ATPase of mitochondria, as well as changing the pectin and cellulose microfibrils in the pollen tube wall. Additionally, the highest level of auxin was found in the pistil when the pollen is germinating, and afterwards significantly decreased levels were found as the tube grew through this section (Chen and Zhao 2008).

The phytohormone auxin plays a prominent role in regulating acclimation to high temperature in plants, and high temperature boosts the level of free auxin (Sun et al. 2012; Wit et al. 2014; Zheng et al. 2016). In *Arabidopsis* seedlings, a moderately high temperature stimulates the elongation of hypocotyls by activation of auxin biosynthesis (Gray et al. 1998). The results of Franklin et al. (2011) indicated that increase levels of auxin were required in an adaptive response to heat stress. However, in the developing anthers of barley and *Arabidopsis*, endogenous auxin levels significantly decreased under heat stress leading to spikelet sterility, and exogenous auxin reversed this damage, suggesting that auxin can alleviate heat-damage in reproductive organs (Sakata et al. 2010). In rice, significantly decreased auxin levels were found in spikelets under heat stress, resulting in abnormal floret differentiation and increase in spikelet degradation (Fu et al. 2015b; Zhang et al. 2017). However, the role of auxin in pollen tube elongation under heat stress has seldom been previously reported. It is reported that pollen tube growth in the styles is strongly inhibited by temperature above 35 °C, and the yield of cotton decreased due to the effect of high temperature during floral bud (square) development (Song et al. 2015). Accordingly, we inferred that changes in auxin level might be involved in abnormal pollen tube growth in the pistil of rice under heat stress, as the auxin levels in the pistil of a cultivar with heat sensitive genotype were significantly decreased under heat stress, and thus the spikelet sterility was significantly increased. Additionally, pretreatment of the flowering plants with NAA could prevent spikelet sterility caused by high temperature stress, suggesting that heat stress-induced disruption of auxin metabolism might be the main factor leading to spikelet sterility by inhibiting pollen tube growth. In this experiment, two rice genotypes with different heat tolerance were subjected to heat stress at anthesis pretreated with NAA and the auxin synthesis inhibitor L-aminooxyphenylpropionic acid (AOPP) (Soeno et al. 2010) to evaluate the role of auxin in pollen tube growth in the pistil and spikelet fertility under heat stress, as well as its underlying mechanism. Also, the relationships among auxin, reactive oxygen species (ROS) and peroxidase (POD) involved in pollen tube growth in the pistil under heat stress will be discussed.

## Methods

### Experimental set-up

Two rice genotypes, namely the Nipponbare (NPB) and its mutant high-temperature susceptible (HTS) genotypes, were used in this study. The HTS semi-rolled leaf mutant was isolated from an ethyl methane sulfonate (EMS)-induced japonica rice NPB mutant bank. This mutant has been selfed for more than nine generations and the rolled-leaf phenotype has been stably expressed in greenhouse and field conditions in Hangzhou, Zhejiang Province China. Rice plants were sown in pots of 10 cm radius and 20 cm height at the China National Rice Research Institute, Hangzhou, China. The rice seeds were soaked in running water for 48 h, then sprout at 37 °C for 24 h. About 20 grains were sown in each pot, and then thinned to four plants when the fifth leaf was emerging. The pots were filled with 7.5 kg of paddy soil. The rice plants were cultivated in the greenhouse with an automatic temperature control system to control the temperature until anthesis (Fu et al. 2016), using the following environmental conditions: 12/12 h photoperiod with natural sunlight, temperature of 30/24 °C, relative humidity of 70/80%, day/night, respectively. At anthesis, the flowering spikelets were immediately sprayed with different concentrations of NAA or AOPP, specifically, 0, 1, 10, 50, and 100 μmol·L^− 1^ of NAA, and 10, 50, and 100 μmol·L^− 1^ of AOPP. About 5–10 min later, they were subjected to heat stress of 40 °C for 2 h. The flowering spikelets in the middle of the panicle (except the superior and inferior spikelets) were labeled with a marking pen after the heat stress ended, and one from each group was collected to determine the auxin levels, pollen germination, pollen tube elongation, ROS level in the pistil, and POD activity, while the others were kept until maturity to determine spikelet fertility.

### Measurement of auxin level and POD activity

The auxin level was determined using 10 mg of frozen pistil sample according to the method of Yang et al. (2001). We used enzyme-linked immunosorbent assay (ELISA) analysis and the procedures were based on the instructions provided by the manufacturer (China Agricultural University, Beijing, China). The POD activity was determined by the method of Li et al. (2011).

### Measurement of pollen germination and pollen tube growth in the pistil

More than 50 flowering spikelets were collected after the heat stress treatment. The pollinated stigmas were fixed in Carnoy’s fixing reagent (by volume: 30% chloroform, 10% acetic acid, and 57% ethanol). The samples were then washed with purified water, incubated in 10 mol·L^− 1^ NaOH for 6–10 min at 56 °C, and then stained in a 0.1% (*w*/*v*) aniline blue solution for 12 h. The pollen tube germination and growth in the pistil were observed and photographed at 350 nm with a fluorescence microscope (DM4000B, Leica, Wetzlar, Germany).

### Measurement of reactive oxygen species (ROS)

For visualization and analysis of ROS in the stigma, the oxidation-sensitive probe DCFH-DA was used, as previously described by Fu et al. (2016). The pollinated stigmas were collected from the flowering spikelets and were immediately incubated with 5 M DCFH-DA. The fluorescence intensity was measured after 30 min incubation with 5 M DCFH-DA using a fluorescence microscope (DM4000B, Leica).

### Quantitative real-time PCR analysis

Pistil from the heat-stress treated plants were frozen in liquid nitrogen and stored at − 80 °C for 2 h, after flowering. Total RNA was extracted from 10 mg of pistil using the TRI pure reagent (Aidlab Biotechnologies, Beijing, China). RNA was converted into first-strand cDNA using the Rever Tra Ace qPCR RT Master Mix (Toyobo, Shanghai, China) using oligo (dT) as the primer. The resultant cDNA was used as a template for quantitative PCR amplification in a Thermal Cycler Dice Real Time System II (Takara Biotechnology, Dalian, China) using SYBR Green I (Toyobo) as a fluorescent reporter. Primers were designed to amplify 150- to 250-bp fragments using the PRIMER5 software (Rozen and Skaletsky 2000). The expression of 6 genes was analyzed; the primers used for qRT-PCR amplification are listed in Additional file 1: Table S1. PCR reaction and detection were performed as described previously (Feng et al. 2013). The 2^−ΔΔCT^method was used to determine the relative gene transcript levels with the mean value of triplicate experiments.

### Spikelet fertility

When completely mature, the labelled spikelet from rice plants in the heat stress treatment and in the control group were sampled. The percentage of unfilled grains was then determined after drying in an oven at 50 °C for 48 h.

### Statistical analyses

The data in this study were processed and analysed with the SPSS 11.5 software (IBM Corp., Armonk, NY, USA). The mean values and standard errors in the figures represent data from three replicate experiments or otherwise noted. The differences between treatments and genotypes were compared using the analysis of variance (ANOVA) least significant difference test at the 5% probability level.

## Results

### Spikelet fertility

**Fig. 1:**
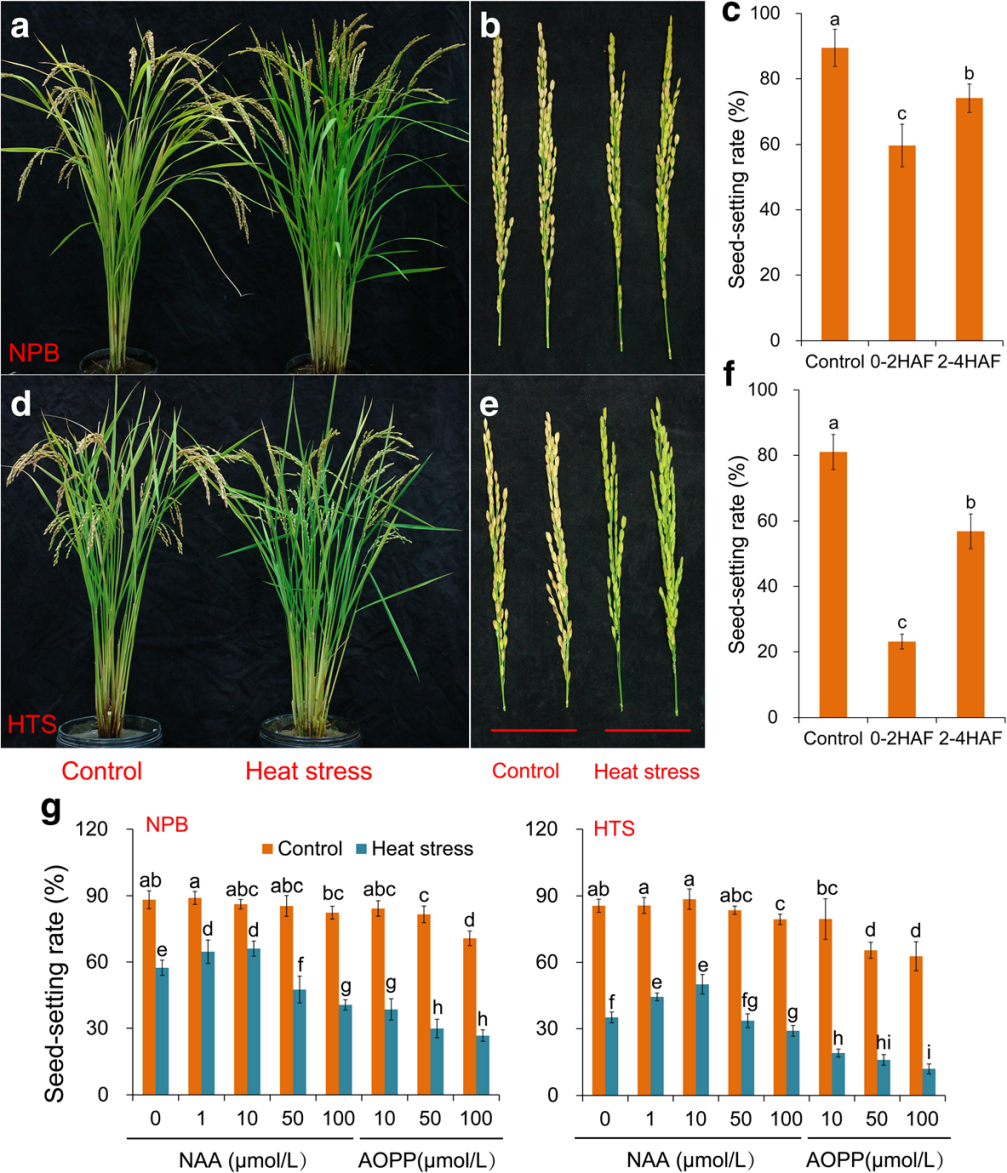
Effect of heat stress on the spikelet fertility of rice plants at the flowering stage. **a**, **b**, **d** and **e** show rice plants with spikelets exposed to heat stress for 10 d at flowering stage. **c** and **f** show the response of spikelet fertility to heat stress at different flowering time period. HAF is hours after flowering. 0–2 HAF, means that the flowering spikelets were immediately subjected to heat stress for 2 h; 2–4 HAF, means that rice plants were subjected in heat stress for 2 h during 2–4 h after flowering. **g** shows the effect of NAA and AOPP on the spikelet fertility of rice plants under heat stress at the flowering stage. Different NAA and AOPP levels were sprayed onto the flowering spikelets before heat stress. Different letters within the same column indicate statistically significant differences between different treatments in the same cultivar (*n* = 3) (*P* < 0.05)

Heat stress at the flowering stage significantly reduced spikelet fertility in rice plants, and the extent of the fertility reduction effect was dependent on the genotypes and heat stress time (Fig. 1a-f). About 33.31% and 17.20% reduction in spikelet fertility was found in the NPB groups receiving the treatment of 0–2 HAF (hours after flowering under heat stress) and 2–4 HAF, respectively compared with the corresponding control group (Fig. 1c), whereas about 71.46% and 29.82% reduction was observed in the HTS groups receiving similar treatments (Fig. 1f). This finding suggested that the treatment of 0–2 HAF had a stronger spikelet fertility reducing effect than the treatment of 2–4 HAF in both genotypes, in which about 19.44% and 59.34% fertility reduction was observed in the NPB group and HTS group, respectively.

We inferred that the disruption of the auxin metabolism caused by heat stress might be the main factor causing spikelet sterility. Accordingly, we sprayed NAA and AOPP onto the rice plants at 0–2 h after flowering to ascertain their role in spikelet sterility (Fig. 1g). Under natural condition, no significant differences in spikelet fertility were found among the NAA treatments in both genotypes except for the 100 μmol·L^− 1^NAA treatment of the HTS cultivar, but it was reduced by AOPP, especially the 100 μmol·L^− 1^ AOPP treatment. Under heat stress, rice plants sprayed with 1 and 10 μmol·L^− 1^ of NAA clearly exhibited increased spikelet fertility in both genotypes compared with that sprayed with H_2_O. Without exception, AOPP treatment significantly decreased the spikelet fertility in both rice genotypes under heat stress, and the spikelet fertility reduction effect of AOPP was concentration dependent. Also, the HTS plants exhibited higher increases in spikelet fertility when sprayed with 1 and 10 μmol·L^− 1^NAA than those of NPB plants compared with that treated with 0 μmol·L^− 1^NAA under heat stress.

### Auxins level and POD activity

**Fig. 2:**
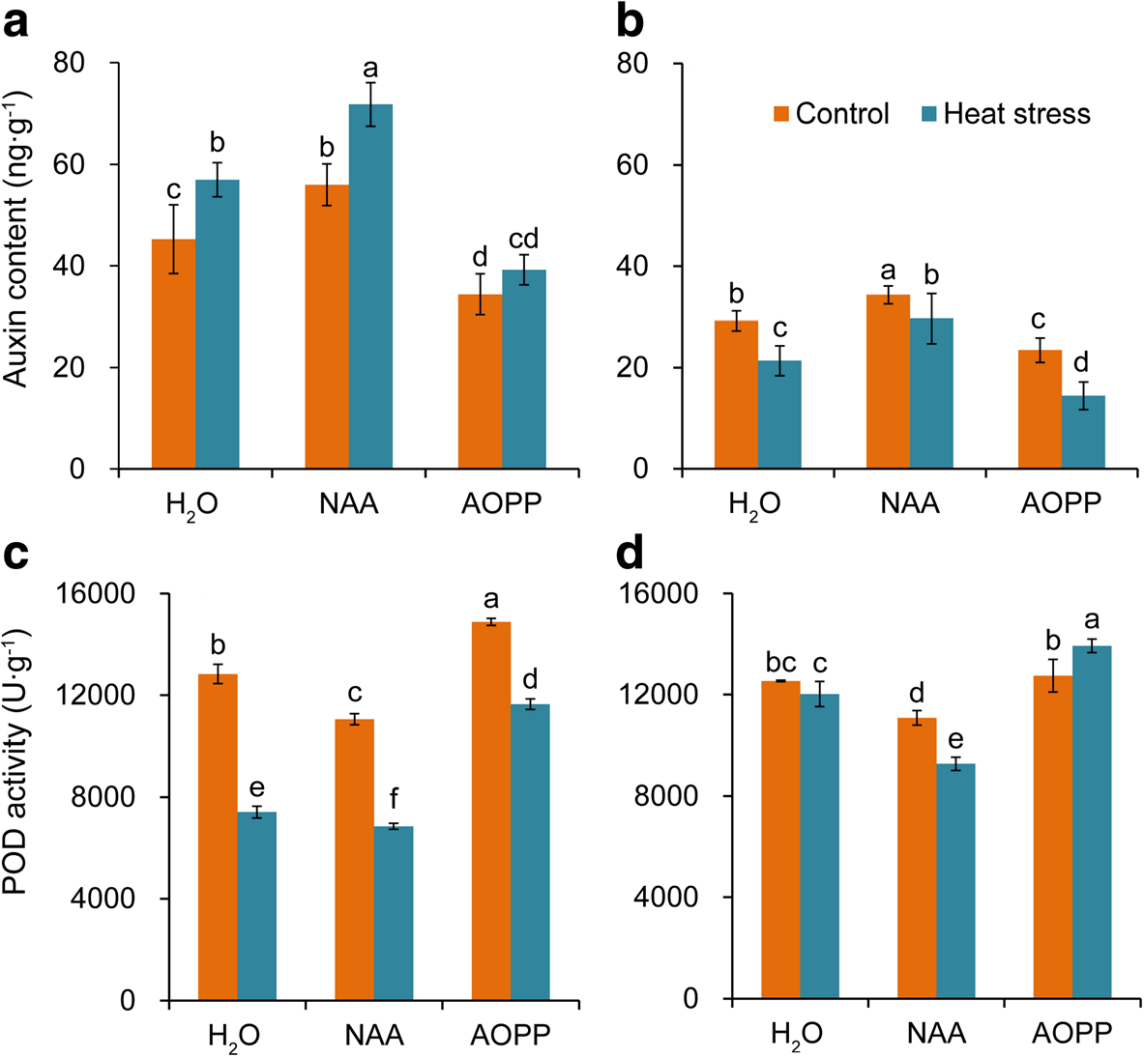
Changes in auxin levels, and POD activities in the pistil under heat stress. Rice plants were sprayed with either 10 μmol·L^− 1^ NAA or 100 μmol·L^− 1^ AOPP under natural and heat-stress conditions. **a** and **b** show the pistil auxin levels in NPB and HTS, respectively; **c** and **d** show the POD activity in the pistil in NPB and HTS, respectively; POD is peroxidase. Vertical bars denote the standard deviation (*n* = 3). Different letters indicate significant differences between control and heat stress treatments (*P* < 0.05)

The treatments with 0, 10 μmol·L^− 1^ NAA and 100 μmol·L^−1^ AOPP were selected to study their effect on the pistil auxin level and POD activity (Fig. 2a-d). Under natural condition, the auxin level was significantly increased in pistils treated with NAA, whereas obvious decrease was found in pistils treated with AOPP, compared with the auxin levels in pistils pretreated with H_2_O (Fig. 2a and b). Under heat stress, the auxins level was significantly increased in the pistils from NPB plants treated with H_2_O and NAA compared with their respective control, whereas little difference was detected between plants subjected to heat stress and control when sprayed with AOPP. Regarding the HTS plants, a sharp decrease in the auxin level was detected in the pistils sprayed with H_2_O under heat stress, whereas a less marked decrease was found with the NAA treatment. There was no doubt that the highest decrease in plants subjected to heat stress was observed with the AOPP treatment.

The POD activity which could suppress auxin by oxidation was significantly decreased in NPB under heat stress, especially with H_2_O and NAA treatments, and the lowest decrease was observed with the AOPP treatment compared with their respective control (Fig. 2c). A slight decrease in the POD activity was observed with the H_2_O treatment of HTS under heat stress (Fig. 2d), and a significantly larger reduction was observed with the NAA treatment. Without exception, an obvious increase was observed with the AOPP treatment under heat stress. Additionally, NAA caused a larger decrease in the levels of POD activity in HTS than in NPB compared with those with H_2_O treatment under heat stress.

### Pollen germination on stigma and pollen tube elongation in pistil

**Fig. 3:**
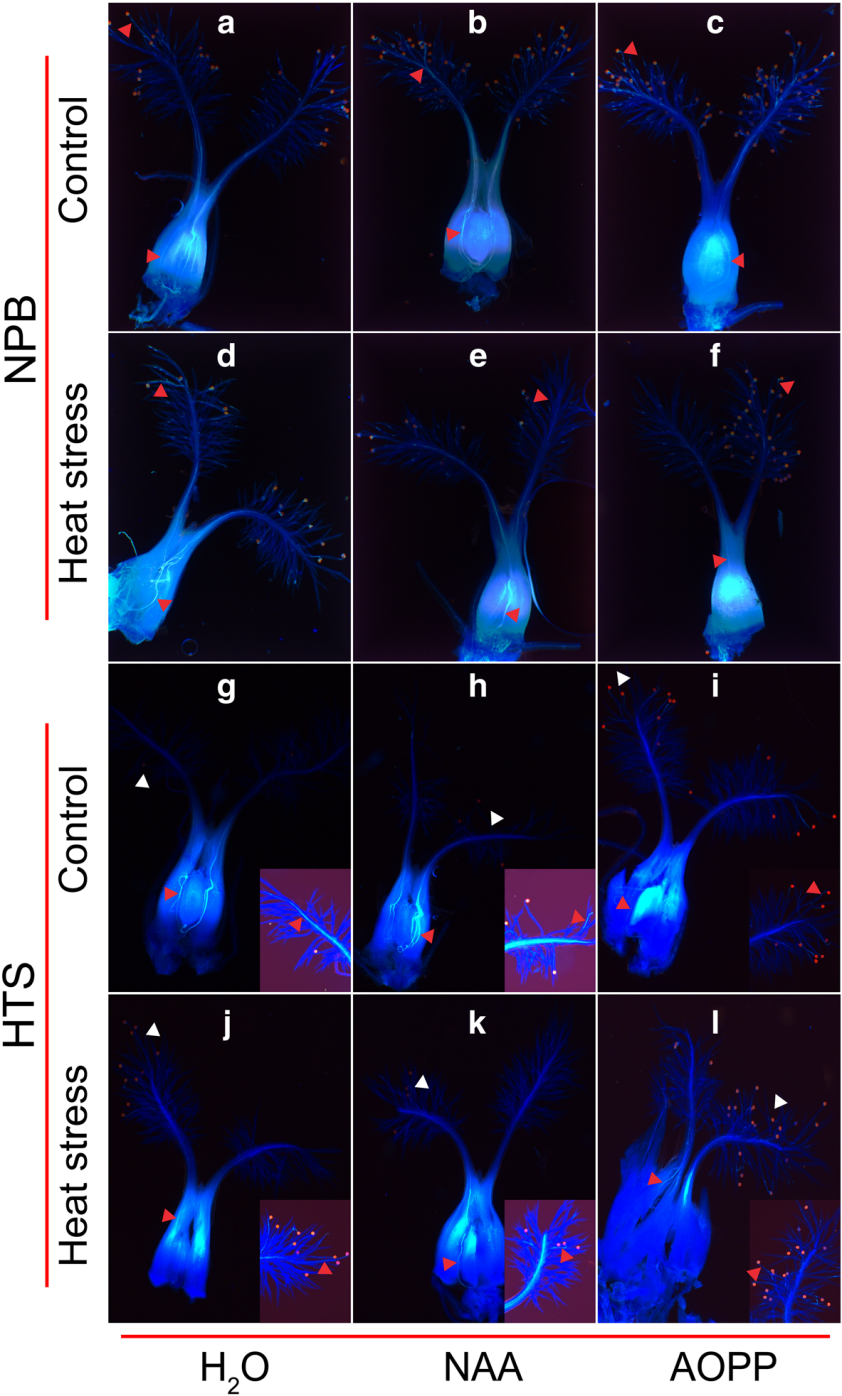
The pollen germination on stigma and pollen tube elongation in rice pistil under heat stress. The fluorescent images displayed in **a**-**c** and **d**-**f** show the NPB pistil with pollen tube under natural and heat-stress conditions, respectively; the fluorescent images displayed in **g**-**i** and **j**-**l** show the HTS pistil with pollen tube under natural and heat stress conditions, respectively. The triangular marks in red indicated the pollen tube; the triangular marks in white indicated the amplifying section in the bottom right of each picture

To study the effect of heat stress on pollen germination and pollen tube elongation, the spikelets were subjected to heat stress about 5–10 min after flowering. No significant differences were found in the pollen germination rate on stigma among all the treatments (data not shown). Under natural condition, the pollen tubes were clearly visible in the style, ovary and ovule of plants of both genotypes, especially with the H_2_O and NAA treatment (Fig. 3a-c and g-i). Under heat stress, the pollen tubes were still visible in the pistil, ovary and ovule of NPB plants with H_2_O and NAA treatment (Fig. 3d and e), whereas the pollen tube was only found in the style of NPB plants with AOPP treatment (Fig. 3f). In addition, the pollen tube was only found in the style of HTS plants with H_2_O and AOPP treatment under heat stress (Fig. 3j and i), and it was clearly visible in the ovary and ovule when sprayed with NAA (Fig. 3k).

### Reactive oxygen species (ROS)

**Fig. 4:**
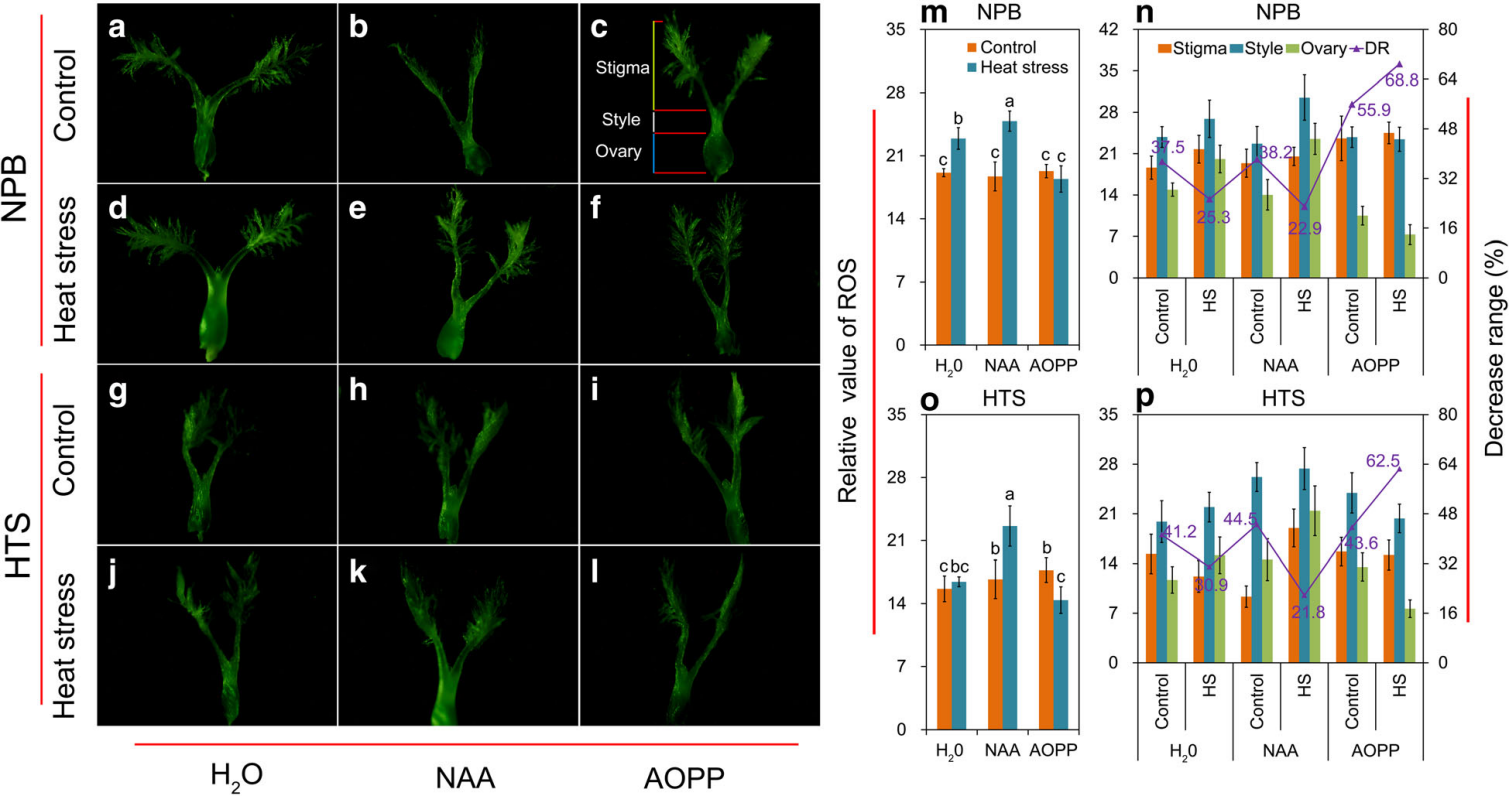
The ROS level in the pistil under heat stress sprayed with NAA or AOPP. **a**-**l** the fluorescent images of pistil in NPB (**a**-**f**) and HTS (**g**-**l**) under natural and heat stress conditions; **m** and **o** show the average ROS levels, under heat-stress and natural conditions, in the NPB pistil and HTS pistil, respectively; **n** and **p** show the relative ROS levels, under heat stress and natural conditions, in the stigma, style and ovary of NPB and HTS, respectively. The levels of ROS in the three components of the pistil, the stigma, style and ovary, were found to be different. Vertical bars denote the standard deviation (*n* = 10). Different letters indicate significant differences between control and heat-stress treatments (*P* < 0.05)

ROS as a molecular signal is involved in the pollen tube growth that can be induced by auxin in plants. The relative levels of ROS were evaluated by using the fluorescence images of the pistils (Fig. 4a-l). Under natural conditions, there was no significant difference in ROS level in the pistil among the various treatments in NPB (Fig. 4a-c and m) and HTS (Fig. 4g-i and 0). Under heat stress, a remarkable increase in ROS level was found in NPB plants with H_2_O and NAA treatment, especially with the latter, whereas a slight decrease was detected with the AOPP treatment compared with their respective control. No significant difference in the ROS level was observed between heat stress and control in HTS plants with H_2_O treatment, whereas significantly increased level of ROS was induced by NAA under heat stress. However, the ROS level was significantly decreased, compared with control, when the plants were sprayed with AOPP under heat stress.

The pistil of rice is composed by the stigma, style and ovary, and different ROS levels were detected in these tissues (Fig. 4c) in NPB (Fig. 4a-c, g-i and m-n) and HTS (Fig. 4d-f, i-l and o-p). The highest ROS level was found in the style, followed by the stigma and ovary in both genotypes; irrespective of whether it was under natural conditions or heat stress (Fig. 4n and p). Compared with the style, under natural conditions, decreases in ROS level of about 37.5, 38.2 and 55.9% were found in the ovary of NPB plants with H_2_O, NAA and AOPP treatments, respectively, while under heat stress the decreases in ROS level were 25.3, 22.9 and 68.8%, respectively (Fig. 4n). For HTS under natural conditions, the decreases in the ROS level were 41.2, 44.5 and 43.6% with H_2_O, NAA and AOPP treatments, respectively, while under heat stress the ROS level decreases were about 30.9, 21.8 and 62.5%, respectively (Fig. 4p).

### Expression levels of *YUC* genes, *rboh* genes and *POX* gene

**Fig. 5:**
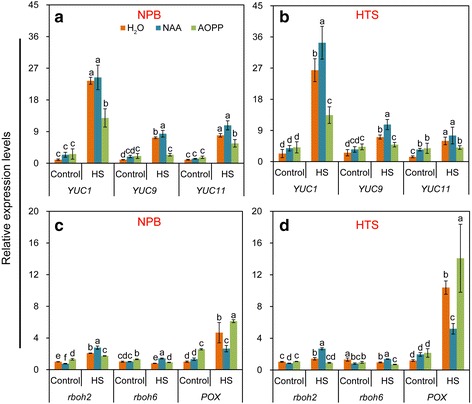
Expression levels of the *YUC* genes, *rboh* genes, and *POX* gene in rice pistil. **a** and **b** *YUC* genes in NPB and HTS, respectively; **c** and **d** *rboh* genes, and *POX* gene in NPB and HTS, respectively*.* Vertical bars denote the standard deviation (*n* = 4). Different letters indicate significant differences between control and heat stress treatments (*P* < 0.05)

The relative expression of six genes, namely *YUC1*, *YUC9*, *YUC11*, *rboh2*, *rboh6,* and *POX,* which might be involved in the changes of the auxin levels, was determined in the pistil (Fig. 5). The *YUC* genes are involved in the auxin biosynthesis pathway, and their expression is associated with the production of auxin in rice plants. A remarkable increase in the relative expression levels of the *YUC* genes was induced by heat stress. Especially *YUC1*, which showed the highest increase in expression in pistils of both genotypes with NAA treatment, and followed by the H_2_O and AOPP treatments in both genotypes (Fig. 5a and b). Compared with HTS, a higher increase in expression induced by heat stress was observed in NPB pistils with H_2_O treatment, whereas when sprayed with NAA the increase in HTS pistils was higher than that in NPB pistils. Similar patterns of expression change were found for the *rboh* genes, which was mainly responsible for the production of ROS (Fig. 5c and d). The relative expression of *rboh* genes was significantly increased by heat stress*,* especially that of *rboh2*. A significantly higher increase in the expression of the *rboh* genes was found in NPB pistils than in HTS pistils with H_2_O treatment, but when sprayed with NAA the increase in the latter was higher than that in the former. In addition, the expression of *POX* gene*,* which was related to the production of POD, was significantly increased by heat stress. The highest increase was found with AOPP treatment, which was significantly higher than those with H_2_O and NAA treatments, compared with their respective controls in both genotypes (Fig. 5c and d). Moreover, compared with HTS, a lower increase in the relative expression of *POX* was found in NPB pistils sprayed with H_2_O under heat stress (Fig. 5c and d). However, a larger decrease was found in HTS pistils sprayed with NAA compared with that in HTS pistils sprayed with H_2_O under heat stress.

## Discussion

It has been well documented that heat stress at the flowering stage can lead to spikelet sterility by inhibiting anther dehiscence, pollen germination on the stigma and pollen tube elongation in the pistil (Snider et al. 2011; Jiang et al. 2015; Coast et al. 2016). Similar results were observed in this study indicating that heat stress caused a sharp decrease in spikelet fertility in these two genotypes compared with their respective control (Fig. 1). However, the extent of fertility reduction effect was found to be dependent on the time of exposure of the flowering spikelet to heat stress (Snider et al. 2011). An evidently higher decrease in spikelet fertility was observed with the 0–2 HAF treatment than that with the 2–4 HAF treatment (Fig. 1), suggesting that the former was the more sensitive to heat stress. The rice pollen is germinated on the stigma within 2 min of pollination, and the tubes reach the ovule after 50 min (Pan 2004; Chen et al. 2008). High temperature stress at this stage not only inhibits the anther dehiscence and pollen germination on the stigma, but also interrupts the pollen tube elongation in the pistils, which has seldom been previously reported (Song et al. 2015). Accordingly, heat stress may inhibit the development of fertilized ovum, rather than pollen tube elongation when the spikelets are subjected to heat stress for about 2 h after flowering.

The cessation of pollen tube growth caused by heat stress might be another important factor resulting in spikelet sterility in rice, especially in high temperature susceptible cultivars, such as the HTS cultivar (Fig. 3). Since the flowering spikelets subjected to heat stress were about 5–10 min later, the spikelet sterility caused by heat stress was due to cessation of pollen tube elongation in the pistil, rather than poor anther dehiscence or lower pollen germination on the stigma (Karapanos et al. 2010; Rang et al. 2011; Djanaguiraman et al. 2013; Song et al. 2015). Indeed, there was no obvious difference in pollen number and pollen germination on the stigma of rice among all the treatments in both genotypes. However, while the pollen tube was invisible in the ovary and ovule of HTS sprayed with H_2_O under heat stress, that of NPB was clearly visible (Fig. 4). Noteworthy, the cessation of pollen tube growth caused by heat stress in the ovary was reversed by NAA, as indicated by the finding that pollen tube elongation under NAA treatment was detected in the ovule in both genotypes, whereas this phenomenon was not observed under AOPP treatment. This finding indicated that the penetration ability of the pollen tube in the pistil under heat stress could lead to the different heat tolerance between these two genotypes, which can be mediated by the auxin level.

The auxin level in the pistil is reported to be significantly increased during fertilization, suggesting that it plays critical roles in pollen germination and pollen tube elongation in the pistil (Wu et al. 2008). The Arabidopsis mutant defective in auxin synthesis impaired the heat tolerance at the pollen mother cell meiosis stage, and exogenous auxin reversed the pollen sterility caused by heat stress (Sakata et al. 2010). These findings are consistent with our results that the auxin level in pistils was significantly increased in NPB, whereas a sharp decrease was found in HTS under heat stress. However, Min et al. (2014) reported that high temperature induced the expression of *CASEIN KINASEI* (*GhCKI*) in heat susceptible cotton, coupled with the suppression of starch synthase activity, decreases in glucose level during anther development and increase in auxin level in late-stage anthers. These findings suggested that high background auxin might be a disadvantage for late stage cotton anthers development during heat stress (Min et al. 2014). Unfortunately, higher auxin level was found in NPB than HTS under natural condition, and under heat stress a remarkable increase was found in the former while a larger decrease were recorded in the latter compared with their respective control under natural conditions (Fig. 2a and b). These different results suggest that the mechanisms underlying the different responses of auxin to heat stress in plants are ambiguous and require further investigation.

Undoubtedly, auxin has very important roles in pollen tube growth in plants, a process during which auxin reaches its highest level in the stigma and is mainly distributed in the transmitting tissue (Chen and Zhao 2008). After the pollen tubes enter the styles, the signal increases in the part where the pollen tubes would enter and then rapidly declines in the part where the pollen tubes have penetrated, suggesting that auxin acts as a molecular signal to guide the pollen tube growing into the sac (Chen and Zhao 2008). Thus, a significant decrease of the auxin level in HTS under heat stress could disrupt pollen tube growth within the pistil, resulting in cessation of pollen tubes growth. Since auxin can attract more energy to for the fertilization in the pistil (Pan et al. 2004), this decrease in the auxin level also disrupt energy homeostasis, resulting in the cessation of pollen tube growth, as this is a high ATP-consuming process (Selinski et al. 2014). It has been reported that not only the mitochondrial respiration and fermentation (Johns et al. 1993; Tadege and Kuhlemeier 1997; Taylor and Hepler 1997), but also plastidial glycolysis and the balancing of the ATP/NAD(P)H ratio contribute to satisfy the energy demand (Cárdenas et al. 2006; Tang et al., 2009; Fujiwara et al. 2010, 2012; Hashida et al. 2013).

The auxin level in the pistil of rice was found to be regulated by NAA in this experiment. Specifically, that the auxin level in the NAA-treated pistil was significantly higher than those treated with H_2_O and AOPP in both genotypes under natural or heat-stress conditions (Fig. 1g and Fig. 3a and b). Accordingly, the relative expression of *YUC* genes were higher with NAA treatment than those with H_2_O and AOPP treatments under heat stress (Fig. 5a and b). These findings were consistent with the results of Wang et al. (2017), who reported that auxin treatment upregulated the expression levels of the several *YUC* genes in bamboo. Similar finding was also found in Arabidopsis (Wang et al. 2017), which after being treated with NAA for 12 h, exhibited a relative expression of *CTL1* that was related to auxin distribution and which was enhanced in the meristem and elongation zones of roots with increasing NAA concentrations. However, Suzuki et al. (2015) reported that NAA decreased endogenous auxin levels and the expression levels of several *YUC* genes in Arabidopsis. We inferred that these different results might be related to the NAA concentrations and the ways of spraying NAA onto plants, rather than the plant species. Indeed, it has been reported that lower concentrations of NAA (< 1 μmol·L^− 1^) enhanced the auxin levels by increasing the relative expression of *YUC* genes *and CTL1* in bamboo and Arabidopsis, respectively (Wang et al. 2017 and b), while higher concentrations (> 5 μmol·L^− 1^) in liquid medium inhibited the auxin level by repressing the expression of *YUC* genes (Suzuki et al. 2015; Wang et al. 2017). Also, in this study, about 10 μmol·L^− 1^ NAA exogenously spayed onto spikelets could reduce the spikelet sterility caused by heat stress at anthesis of rice, which was consistent with the results of Sakata et al. (2010) in Arabidopsis accompanied with the enhancement in auxin levels and the relative expression of *YUC* genes.

ROS is needed to sustain the normal rate of pollen tube growth and this is likely to be a general mechanism in the control of the growth of the tip of polarized plant cells (Potocký et al. 2007), which suggests that ROS is involved in auxin-induced pollen tube growth in the pistil of rice under heat stress. The results of the present study revealed a remarkable increase in ROS induced by heat stress in NPB with H_2_O treatment, while no obvious difference was found in HTS compared with the control (Fig. 4). Also, the ROS was significantly increased in both genotypes when sprayed with NAA, particularly in HTS. In contrast, inhibition of ROS production was observed in plants with AOPP treatments, which suggested that crosstalk between auxin and ROS occur in plants under abiotic stress (Joo et al. 2001; Song et al. 2006). Auxin induces apoplastic superoxide ion production, which facilitates the loosening of the cell wall during cell elongation and growth in maize and *Arabidopsis thaliana* (Schopfer et al. 2001; Mangano et al. 2017). In turn, the increased ROS level affects auxin gradients and/or sensitivity, including the oxidative degradation of auxin and auxin transport and distribution (Kawano et al. 2003). In fact, non-uniform distribution of ROS was shown in the pistil with the highest level found in the style, followed by the stigma and ovary in both genotypes irrespective of whether it was under natural condition or heat stress (Fig. 4). Thus, the changes in ROS level in the ovary might play a more important role in the elongation of the pollen tube into the ovule, as it could lead to the formation of an auxin gradient in the pistil during pollination. The results presented here showed that the ROS level was lower in the ovary compared with that in the style in both genotypes under heat stress compared with those under natural conditions. Accordingly, a larger decrease was found in HTS than in NPB with H_2_O treatment. However, the decrease in ROS level in NPB was smaller than that in HTS in the presence of NAA compared with those with H_2_O treatment under heat stress (Fig. 4n and p). However, similar results have not been documented in abiotic stress, suggesting that the role of a ROS gradient in the pistil in pollen tube elongation remains ambiguous.

POD is important for plants to survive under abiotic stress by scavenging excess ROS to maintain redox homeostasis (Zhang et al. 2017). However, POD is also an auxin oxidase that reduces auxin in plants (Mathesius et al. 2001). Accordingly, we inferred that POD might negatively mediate pollen tube elongation in pistils under heat stress, since the highest increase was found with the AOPP treatment in both genotypes under heat stress, followed by the treatment with H_2_O and NAA compared with their respective control (Fig. 2c and d). This finding suggested that NAA can reduce the POD activity in the pistil under heat stress by inhibiting the relative expression level of *POX* (Fig. 5c and d), which has seldom been previously reported. Additionally, under heat stress, significantly higher decrease in POD activity was found in NPB plants under H_2_O treatment compared with its respective control, while no significant difference was found in HTS plants (Fig. 2c and d), which indicated that POD might be the main factor leading to the different heat tolerance between these two genotypes.

**Fig. 6:**
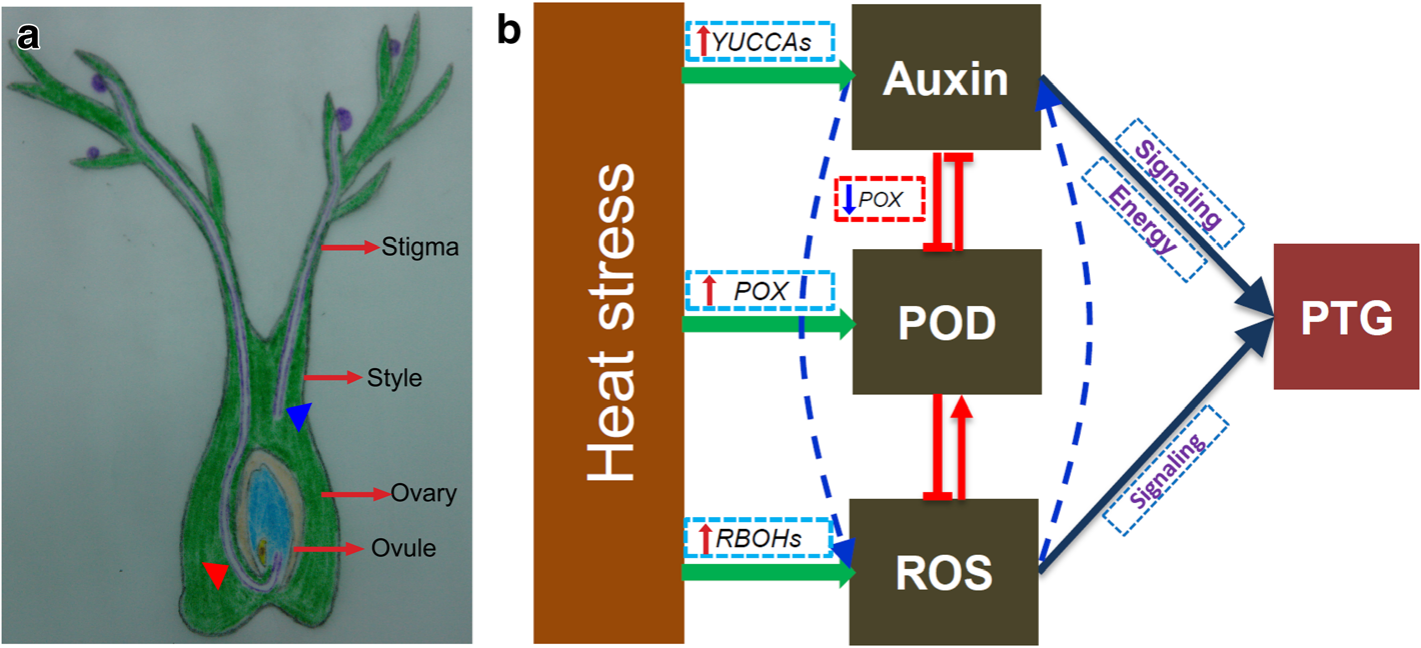
Descriptive model of the function of auxin in the pollen tube growth in rice pistil. The level of auxin, ROS and POD in the pistil are obviously affected by heat stress, and thus the relationships among them are involved in the pollen tube elongation in the pistil. The triangle symbol indicates the pollen tube in the pistils, while the triangle in red indicates the pollen tube under natural conditions, and that the green triangle indicates the ceasing of pollen tube growth in the style under heat stress. PTG, denotes pollen tube growth. The pointed arrow , indicates enhancement, while the blunt-ended arrow , indicates inhibition

According to the above discussion, under natural conditions the pollen tubes grow in the pistil, and then elongate into the ovule under natural conditions, whereas under heat stress they cease to grow in the stigma or style before reaching the ovule (Fig. 6a). Auxin plays a key role in pollen tube elongation in the pistil by acting as a guiding signal and providing sufficient energy, as it is a high energy-demanding process (Fig. 6b). ROS also acts as signaling molecules in pollen tube elongation in the pistil, and the crosstalk between ROS and auxin are also involved in this process, as indicated by the similarity of their patterns of change under heat stress. POD negatively regulates pollen tube elongation by degrading auxin and scavenging ROS. Under heat stress, the POD activity was significantly decreased in NPB, while no significant change in POD activity was found in HTS compared with control, suggesting that it might be the main factor leading to the decrease in auxin and ROS. During this process, exogenous NAA can reduce the POD activity by inhibiting the relative expression level of *POX*, while ROS can induce an increase of the POD activity.

## Conclusion

Heat stress at anthesis significantly reduces the spikelet sterility of rice, which can be prevented by exogenous treatment with NAA, especially in the high temperature susceptible line HTS. Larger decrease in the level of auxin was found in HTS than NPB compared with their respective control, thus lower decrease in POD activity was found in the former than the latter. There was no significant difference in pollen tube growth in the pistil, specifically in the stigma, style and ovary, among all the treatments in both genotypes, under natural conditions. The pollen tube in the NPB pistils under H_2_O treatment was still found in the ovary under heat stress, while that in the HTS pistils it was invisible. Also, the pollen tube elongation was observed in the ovary and ovule in both genotypes when sprayed with NAA, but with the AOPP treatment it was not observed under heat stress. This finding suggested that NAA can enhance heat tolerance in plants by preventing the cessation of pollen tube elongation in the pistil caused by heat stress. In fact, similar patterns of change were found in the levels of auxin and ROS in the pistil of both genotypes, indicating that larger increase in auxin and ROS levels were obtained with NAA treatment, followed by treatment with H_2_O and AOPP under heat stress compared with their respective control. However, the highest POD activity was observed with the AOPP treatment, followed by the treatment with H_2_O, and NAA in both genotypes under heat stress. These finding indicated that crosstalk between ROS and auxin might occur during pollen tube elongation in the pistil under heat stress, while POD might be the factor negatively affecting this process (Fig. 6).

## Additional file

Additional file 1: **Table S1.** Primer sequences used in qRT-PCR analysis. (DOCX 33 kb)

## Abbreviations

AOPP: L-aminooxyphenylpropionic acid
ELISA: Enzyme-linked immunosorbent assay
HTS: High temperature susceptible
IAA: Indole-3-acetic acid
NAA: 1-naphthaleneacetic acid
NPA: 1-N-naphthylphthalamic acid
NPB: Nipponbare
POD: Peroxidase
ROS: Reactive oxygen species
