# Supplementary material for: Heat stress induces spikelet sterility in rice at anthesis through inhibition of pollen tube elongation interfering with auxin homeostasis in pollinated pistils
Source: Rice (N Y). 2018 Mar 12;11:14. doi: 10.1186/s12284-018-0206-5 (PMC5847639; doi:10.1186/s12284-018-0206-5)
Supplement: Supplementary file 1 — Table S1. Primer sequences used in qRT-PCR analysis. (DOCX 33 kb) [file 12284_2018_206_MOESM1_ESM.docx]

**Additional file**

Supplement Table 1 Primer sequences used in qRT-PCR analysis.

| Gene | Accession | Forward (5’-3’) | Reverse (5’-3’) |
| --- | --- | --- | --- |
| *YUC1* | LOC_Os01g45760 | TGTAGGATGCCGGTGTCTTGTT | TCGGTCCCTTTCTCTCCATTGG |
| *YUC9* | LOC_Os01g16714 | AAGAGTGATGACGGGCTGATCG | GGAGTTGCTTGCTGATGTCGTC |
| *YUC11* | LOC_Os12g08780 | GTTCTGATTGTTGGAGCAGGCC | CTTTGCGAGGTGAAGCTTGAGG |
| *POX* | LOC_Os01g15830 | CTCCCCACCATCATCACCAAGT | AGTGTGTAGTCAGCCATGCCAT |
| *rboh2* | LOC_Os01g53294 | CCAGGTCAGCACTCATCACCAT | TTGGTCGTGCAAAATGTGTCCG |
| *rboh6* | LOC_Os08g35210 | CAGGGCACACTTCTACTGGGTT | TGTGCAGCTCTATGACACCCTT |
| *UBQ5* | LOC_Os01g22490 | GACTACAACATCCAGAAGGAGTC | TCATCTAATAACCAGTTCGATTTC |
